# Supplementary material for: Structure of the Nipah virus polymerase complex
Source: EMBO J. 2024 Dec 30;44(2):563–86. doi: 10.1038/s44318-024-00321-z (PMC11730344; doi:10.1038/s44318-024-00321-z)
Supplement: Supplementary file 1 — Appendix [file 44318_2024_321_MOESM1_ESM.pdf]

Appendix for: Structure of the Nipah virus polymerase complex

Appendix Figures 2

Appendix Figure S1 5

Appendix Figure S2 6

Appendix Figure S3 7

Appendix Figure S4 8

Appendix Figure S5 9

Appendix Figure S6 10

Appendix Figure S7 11

Appendix Figure S8 13

Appendix Figure S9 15

Appendix Table S1 16

Appendix Table S2 17

Appendix Figures

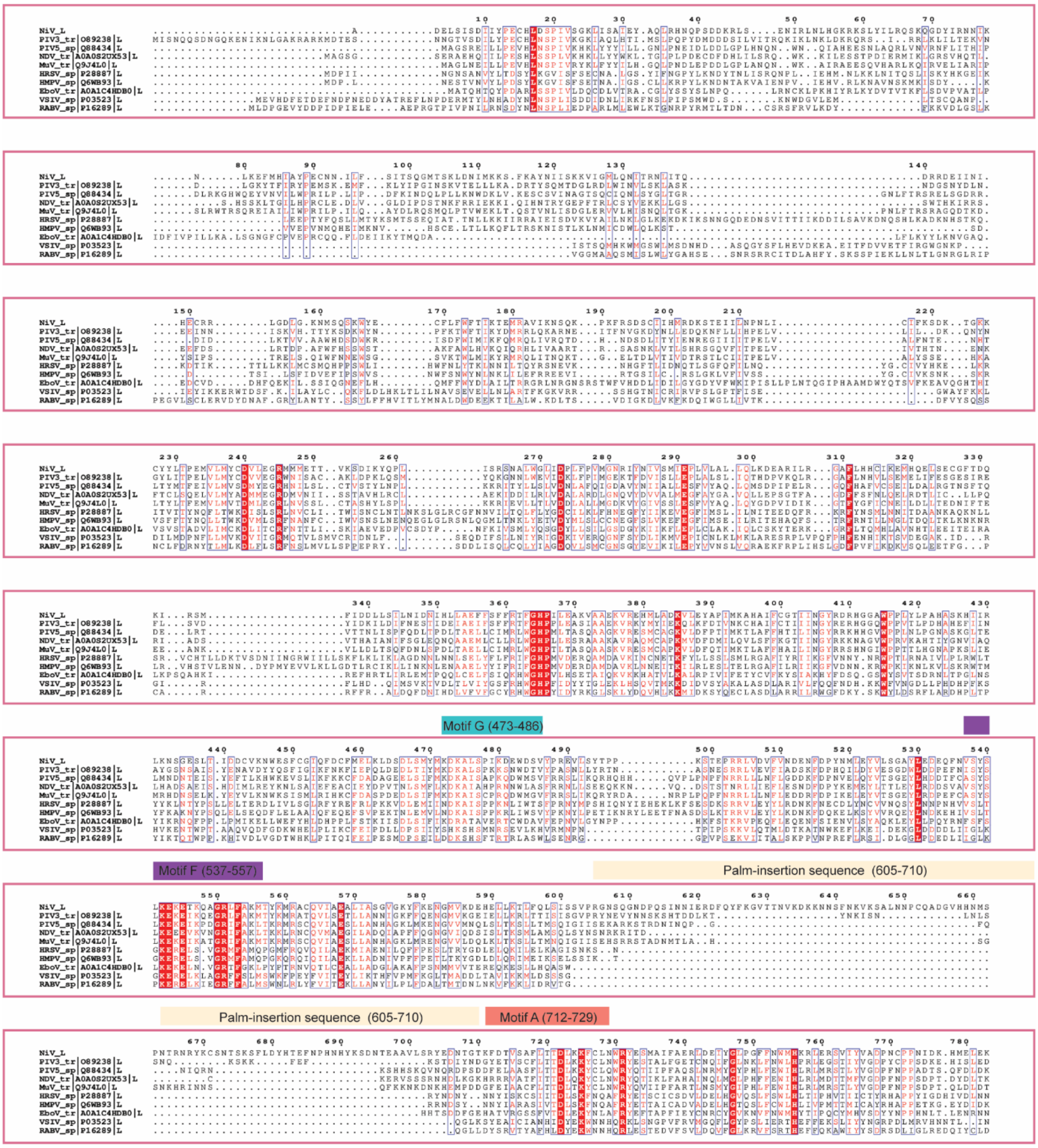
NiV_L
PIV3_tr|O89238|L
PIV5_sp|Q88434|L
NDV_tr|A0A0S2UX53|L
MuV_tr|Q9J4L0|L
HRSV_sp|P28887|L
HMPV_sp|Q6WB93|L
EboV_tr|A0A1C4HDB0|L
VSIV_sp|P03523|L
RABV_sp|P16289|L
Motif G (473-486)
Motif F (537-557)
Palm-insertion sequence (605-710)
Motif A (712-729)

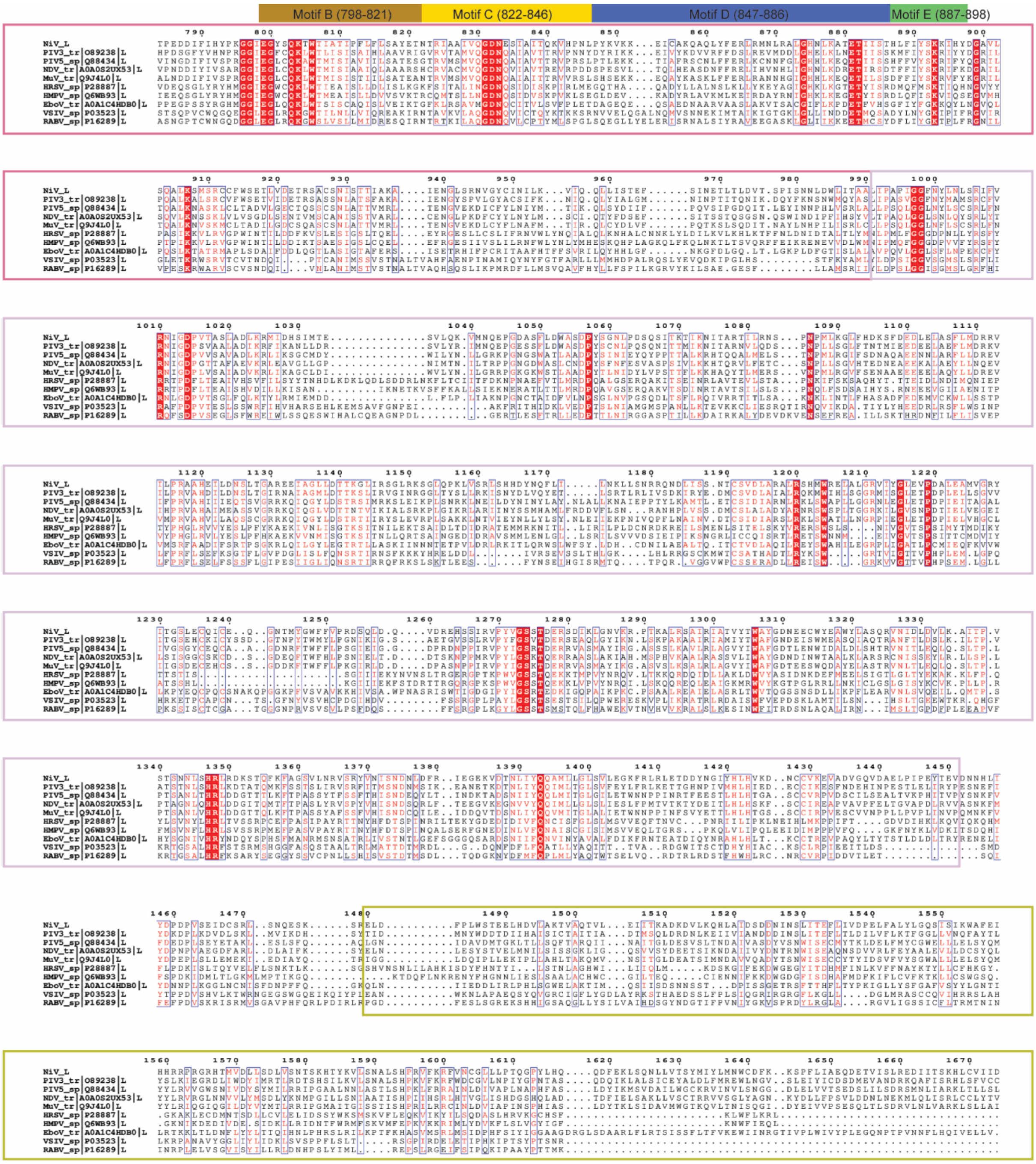
Motif B (798-821)
Motif C (822-846)
Motif D (847-886)
Motif E (887-898)
NiV_L
PIV3_tr|O89238|L
PIV5_sp|Q88434|L
NDV_tr|A0A0S2UX53|L
MuV_tr|Q9J4L0|L
HRSV_sp|P28887|L
HMPV_sp|Q6WB93|L
EboV_tr|A0A1C4HDB0|L
VSIV_sp|P03523|L
RABV_sp|P16289|L

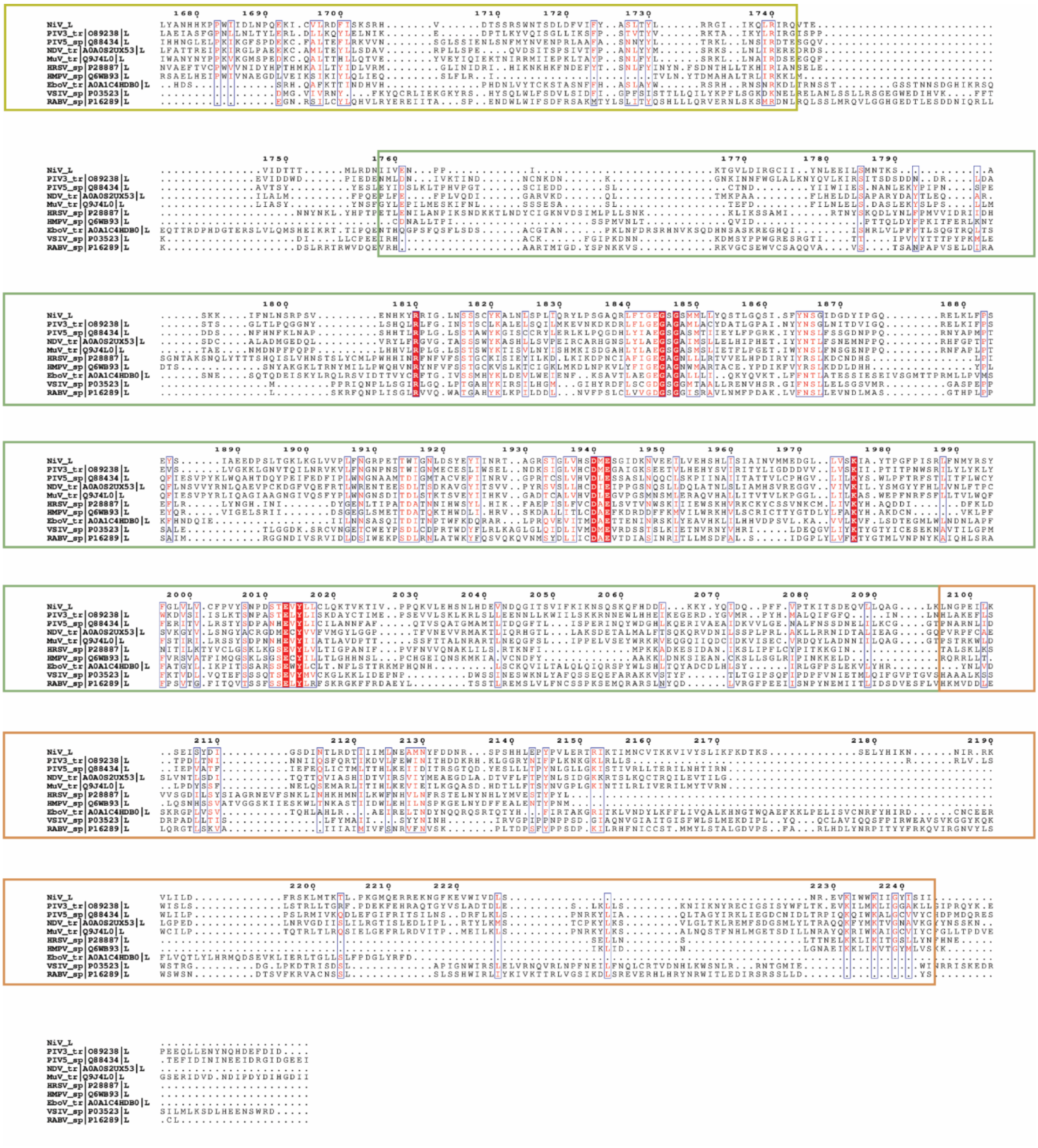

Appendix Figure S1. Multiple sequence alignment for the L-proteins of members of *Paramyxoviridae*, *Pneumoviridae, Filoviridae*, and *Rhabdoviridae*. The RdRp-domain sequence is framed in pale purple red, the PRNTase-domain in pale pink, the CD-domain in yellow, the MTase-domain in green, and the CTD-domain in orange. The palm insertion sequence and the RdRp-domain motifs are represented by bars colored with their respective colours. The amino acid sequences of L-protein from PIV3 (UniProt ID: O89238), PIV5 (UniProt ID: Q88434), NDV (UniProt ID: AOA0S2UX53), MuV (UniProt ID: Q9J4L0), HRSV (UniProt ID: P28887), HMPV (UniProt ID: Q6WB93), EboV (UniProt ID: A0A1C4HDB0), VSIV (UniProt ID: P03523), and RABV (UniProt ID: P16289) were aligned using Clustal Omega and rendered in ESPript 3.0.

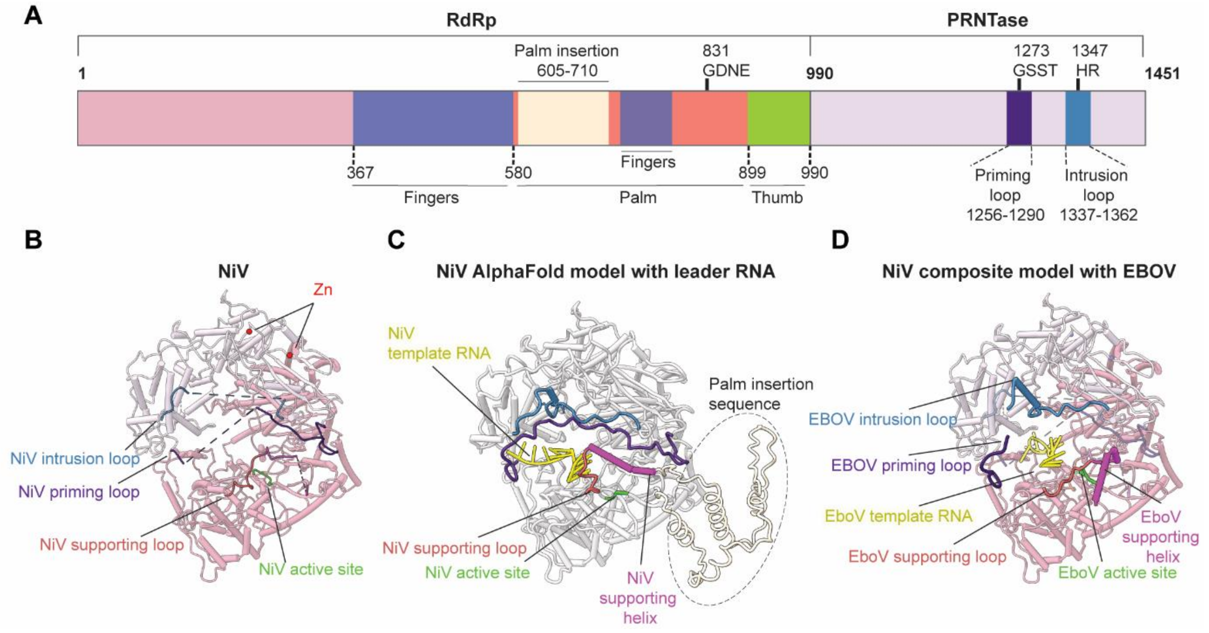

Appendix Figure S2.

A. Schematic diagram of the domain architecture of RdRp-domain of NiV L-protein (pale purple red) and PRNTase-domain (pale pink). The conserved motifs on the RdRp-domain, are represented in different colours as well as the priming and intrusion loops on the PRNTase-domain.

B. The structural elements on the NiV RdRp-domain are represented by arrows. Two zinc ions bound on the NiV PRNTase-domain are represented as red spheres.

C. The NiV L-P complex bound to RNA (5'-CCCUUGUUUGGU-3') was predicted using AlphaFold 3, which demonstrated the stabilization of these structural elements. The predicted region for the palm insertion sequence (residues 605-710) is indicated by a dashed circle.

D. A composite model was generated using the EBOV priming loop, intrusion loop, supporting loop and supporting helix along with its bound template RNA (PDB ID 8JSL).

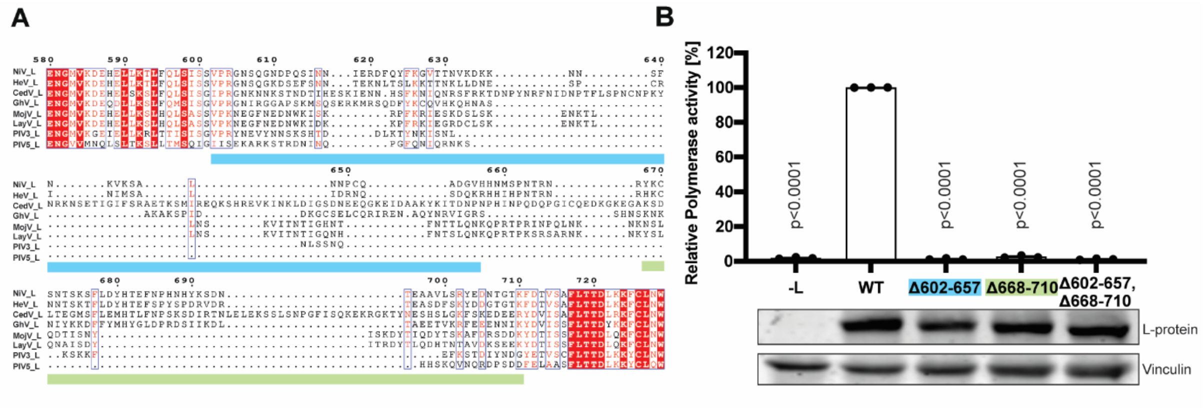

Appendix Figure S3. Henipavirus specific palm insertion sequence

A. Sequence alignment of the palm-insertion sequence region of L-proteins from Henipaviruses and its comparison with the L-proteins of PIV3 and PIV5. The GenBank accession codes for each L-protein of the Henipavirus family are shown alongside the sequence name: QYC64606.1 for Hendra virus, YP_009094087.1 for Cedar virus, YP_009091839.1 for Ghana virus, YP_009094096.1 for Mojiang virus, LUUV47242 for Langya virus. The UniProt IDs are O89238 for PIV3 and Q88434 for PIV5. Sequences were aligned in Clustal Omega and rendered in ESPript 3.0.

B. Polymerase activity dependent on the presence of the NiV L-protein palm insertion assessed using the minireplicon assay. BSR-T7/5 cells were co-transfected with plasmids expressing P-, N- as well as wild type (WT) and truncated versions of L- proteins, together with a minigenome encoding for the Gaussia luciferase. Relative Gaussia luciferase activities are presented as the mean percentage activity of WT (±SEM), n=3 independent biological replicates of technical duplicates. P values from Dunnett's multiple comparison (WT) one-way ANOVA test are indicated. For evaluating the expression levels of L-proteins, HEK-293T cells were transfected with pCAGGs plasmids expressing for wild type or truncated versions of L-protein. At 24 hours p. t., expression levels were analysed by western blot using the indicated antibodies. Representative blots from one experiment are shown.

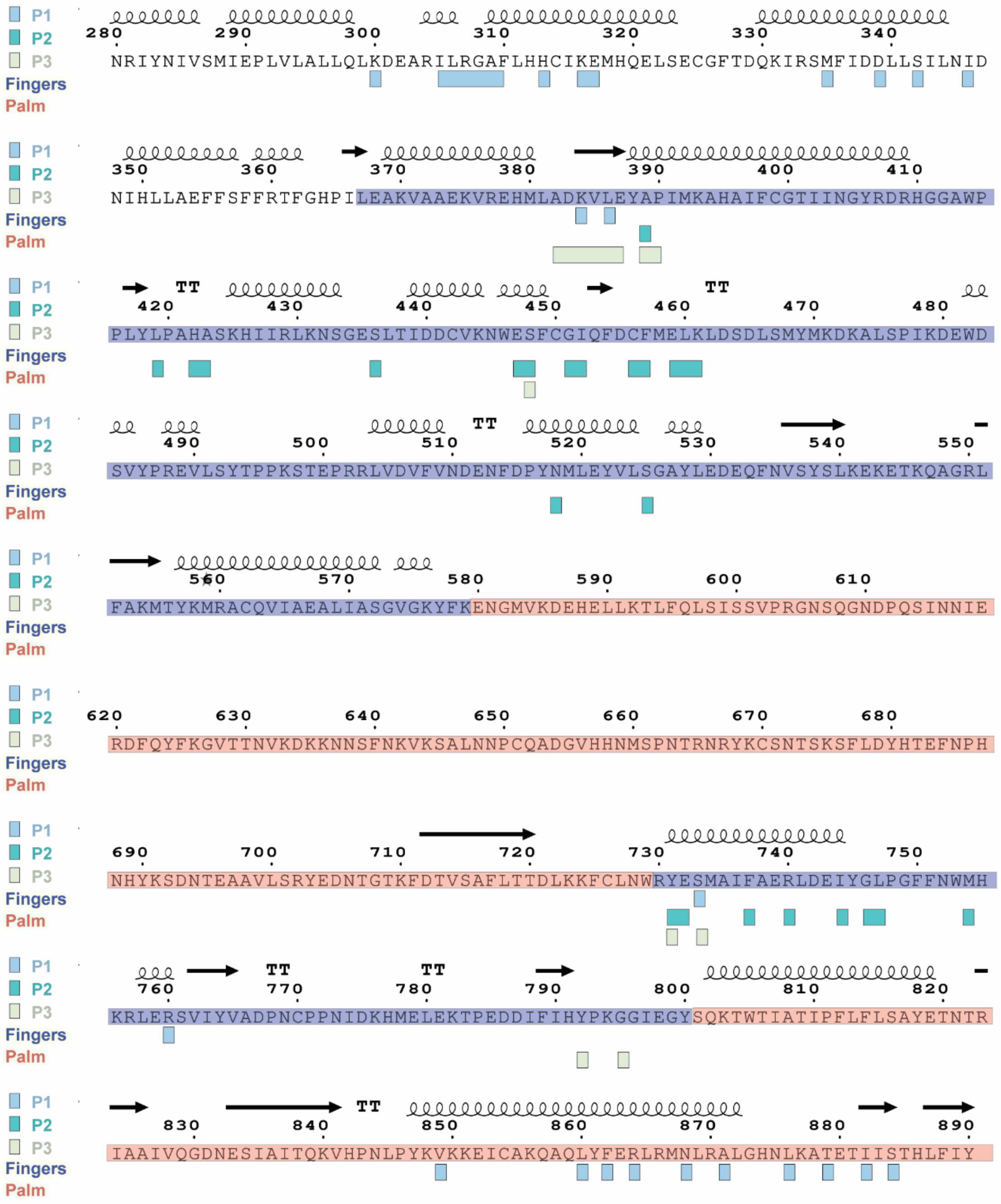

Appendix Figure S4. Binding regions of P-protein protomers on the sequence of L-protein. Each monomer of the P-protein tetramer interacts with various residues on the L-protein, and some of these interactions also involve common regions ((384-389) and (731-733)) on the L-protein sequence that can simultaneously recruit each of the 3 protomers of P-protein.

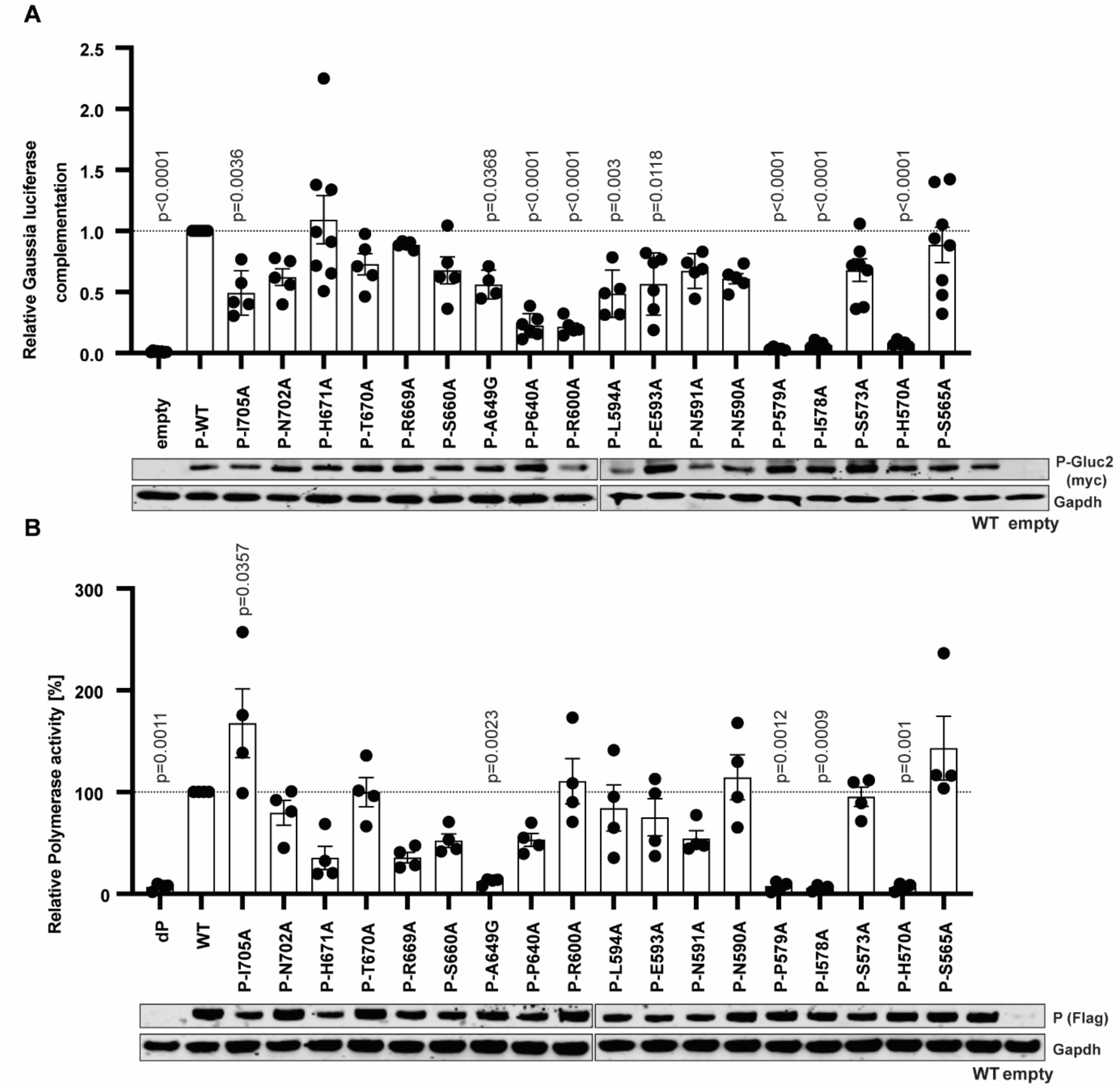

Appendix Figure S5. Role of residues involved in L-P interactions.

A. Split-Luciferase complementation assay was performed to assess the impact of point mutations in the L-P interface on L-P interaction. HEK-293T cells were transfected with plasmids expressing L- and P- proteins (wild type or mutant) fused to Gluc1 or Gluc2, respectively. For negative controls, a Gluc2-empty plasmid was transfected. Relative Gaussia luciferase activities are presented as the mean percentage activity of WT (±SEM), n≥5 independent biological replicates of technical triplicates.

B. Minireplicon assay was performed to investigate the effect of point mutations in the L-P interface on polymerase activity. BSR-T7/5 cells were transfected with plasmids encoding L-, N- and P-proteins (wild type or mutant), together with a minigenome encoding for the Gaussia luciferase. Relative Gaussia luciferase activities are presented as the mean percentage activity of WT (±SEM), n=4 independent biological replicates of technical triplicates. P values

from Dunnett's multiple comparison (WT) one-way ANOVA test are indicated. For evaluating the expression levels of P-proteins (wild type or mutant), HEK-293T cells were transfected with respective plasmids encoding for P-protein fused to a tandem-affinity purification Tag (TAP; Strep-Strep-Flag) or P-protein fused to Gluc2 (N-Terminus) and myc-Tag (C-Terminus). At 24 hours p. t., expression levels were analysed by western blot using the indicated antibodies.

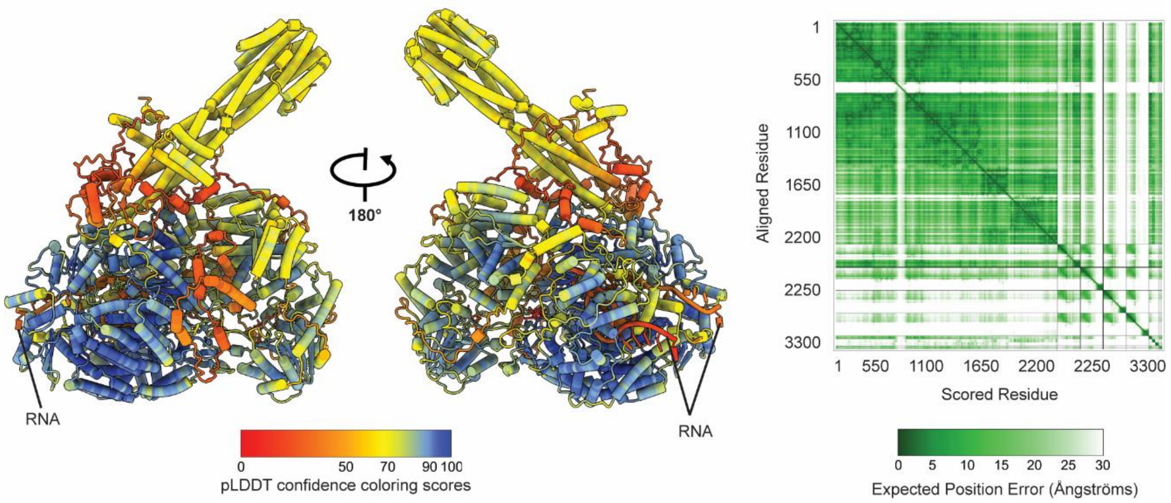

Appendix Figure S6. AF3 model predictions. The AF3 predicted model of the RNA bound NiV L-P complex is colored based on pLDDT confidence scores, with red indicating the lowest confidence and blue indicating the highest confidence. Predicted Aligned Error (PAE) plot for the same prediction in the right side. The plot is colored in shades of green, where darker green indicates higher confidence and lower error, and lighter green represents lower confidence and higher error. The ipTM score of the NiV model is 0.68, while pTM score is 0.76.

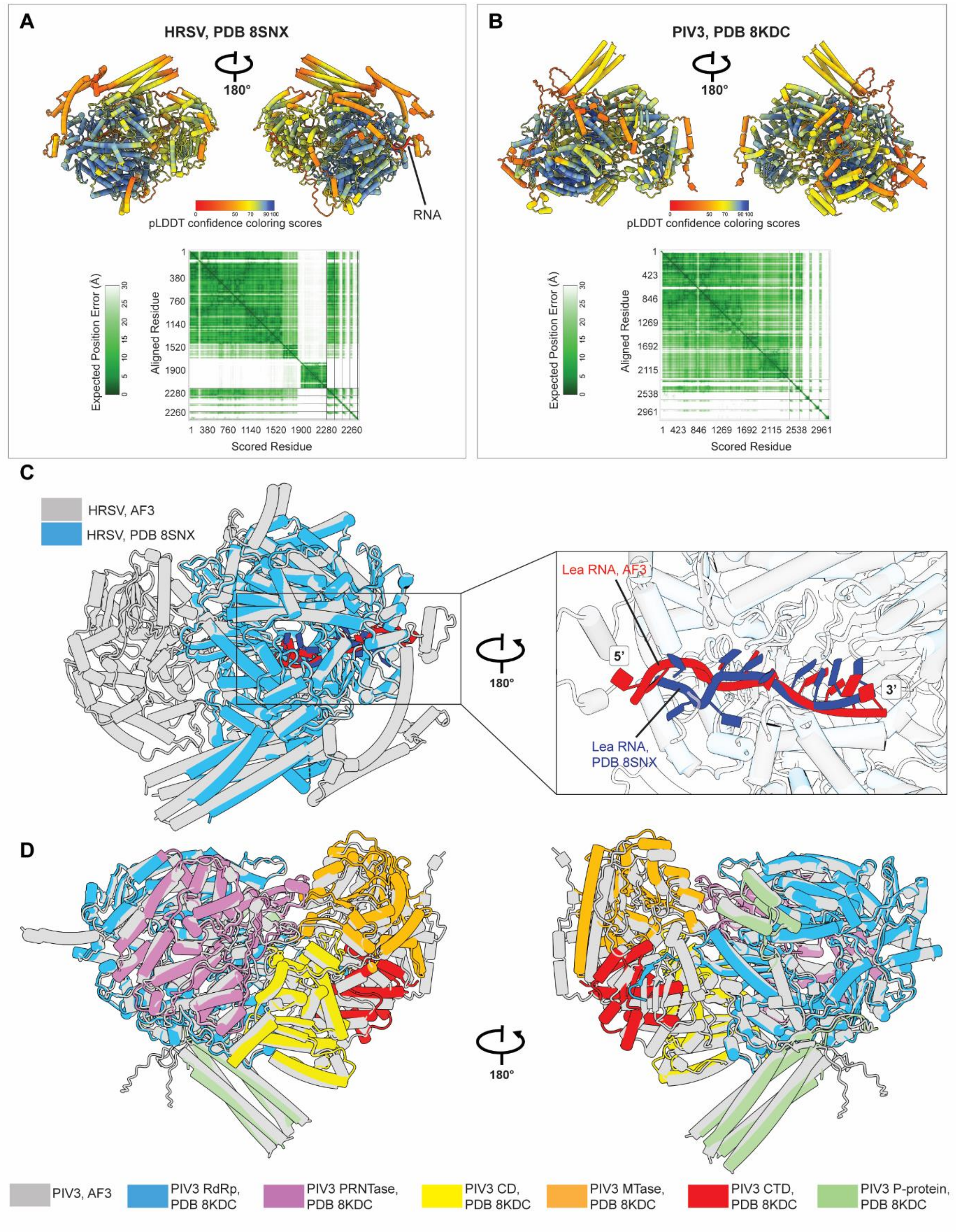

Appendix Figure S7. AF3 model predictions of structurally determined RNA bound L P complexes and L P complexes with modelled CD, MTase, and CTD domains. For the prediction of the RNA bound HRSV structure, we used the sequences and ions based on PDB ID: 8SNX. The full length sequence of HRSV L (UniProt ID: P28887) and four copies of HRSV P (residues 120 241) (UniProt ID: P03421) were used, along with the RNA sequence

UUUUUCGCGU 3'. For the PIV3 structure prediction based on PDB ID: 8KDC, we used the full length PIV3 L sequence (UniProt ID: O89238) and four copies of the PIV3 P sequence (residues 430 603) (UniProt ID: O89234). Additionally, two Zn ions and one Mg ion were included in the prediction.

A. The RNA bound structure of the HRSV complex is shown, with colouring based on pLDDT scores. The related PAE plot is presented at the bottom of the panel, with ipTM = 0.58 and pTM = 0.69.

B. The structure prediction for the PIV3 L P complex is shown, coloured by pLDDT confidence scores. The corresponding PAE plot is displayed at the bottom of the panel, with ipTM = 0.61 and pTM = 0.72.

C. Superimposition of the predicted RNA--bound HRSV L--P complex and the experimentally validated structure (PDB 8SNX), with RMSD of 0.581 Å. A close--up view of the RNA--binding site shows that the predicted RNA and the experimental structure exhibit highly similar binding modes.

D. Superimposition of the predicted PIV3 L--P complex and the experimentally resolved structure (PDB 8KDC), demonstrating close alignment of the predicted domain positions with the solved structure. The RMSD for the superimposition is 1.083 Å.

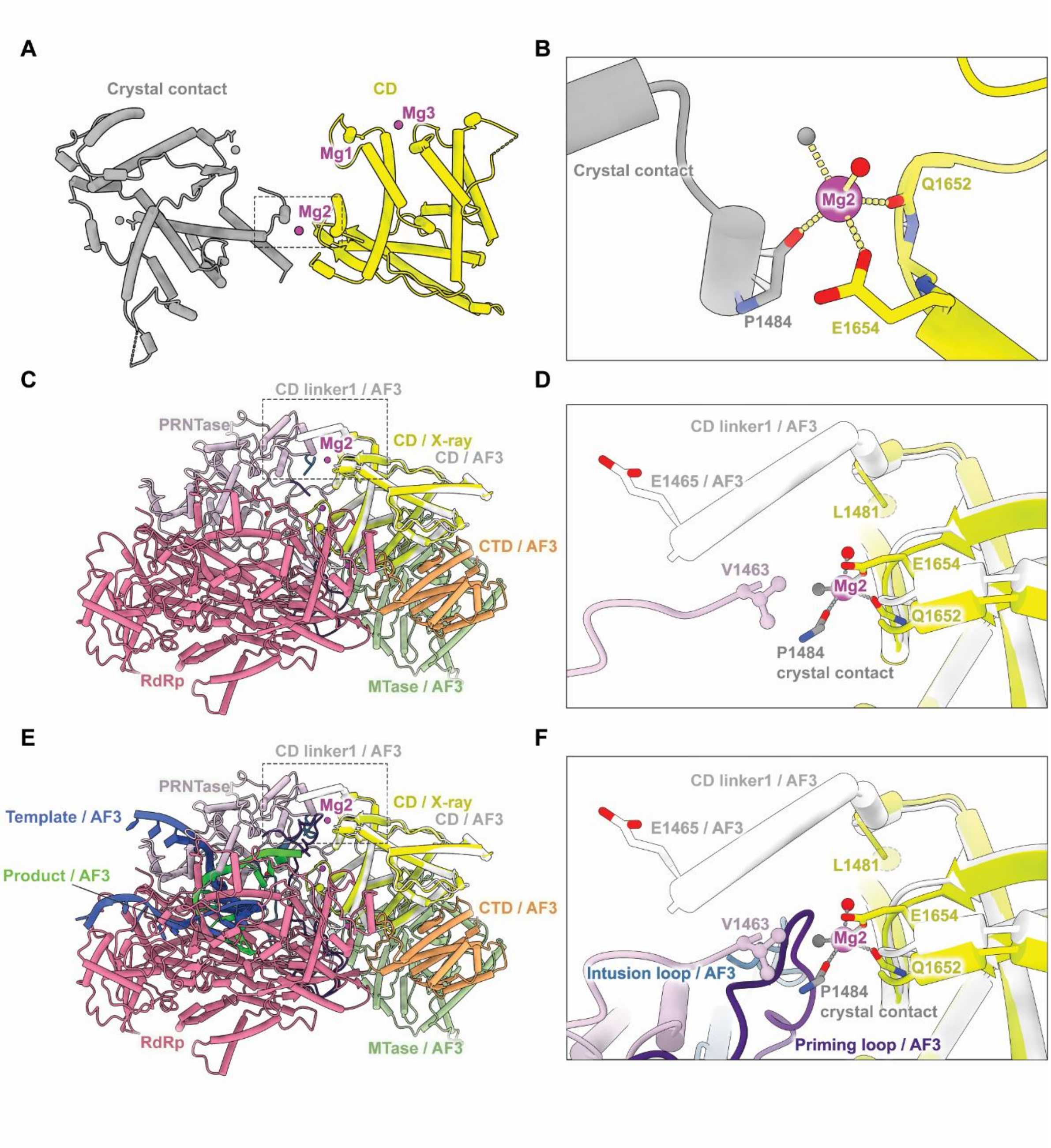

Appendix Figure S8. The crystal structure of the CD-domain bound to three Mg ions (Mg1, Mg2, Mg3) is shown with the Mg ions represented as magenta spheres.

A. Mg2 is coordinated within the CD-domain crystal structure (yellow), along with asymmetrical crystal contacts (grey). Water molecules are depicted as red spheres for the CD-domain and grey spheres for the crystal contacts.

B. The coordination of the Mg2 involves the main chain of Gln1652, the side chain of Glu1654, and a water molecule (red), as well as the main chain of Pro1484 and a water molecule derived from the crystal contact.

C. The position of Mg2 is shown in the composite model comprising RdRp, PRNTase, CD, CD/AF3, MTase/AF3, and CTD/AF3.

D. A close-up view of the bound Mg2 within the composite model is presented, with the coordinating residues depicted as sticks. In the cryo-EM structure of NiV L-protein, the last residue (Val1463) is represented as ball-and-stick, whereas the first residue (Leu1481) built in the CD-domain X-ray structure is shown with dashes. The unbuilt CD linker1, predicted by AF3, is illustrated in white, with residue Glu1465 shown as a stick.

E. The composite model was expanded to include priming loop

F. A close-up view of the RNA-modelled composite reveals the positioning of the Mg2 near the priming loop.

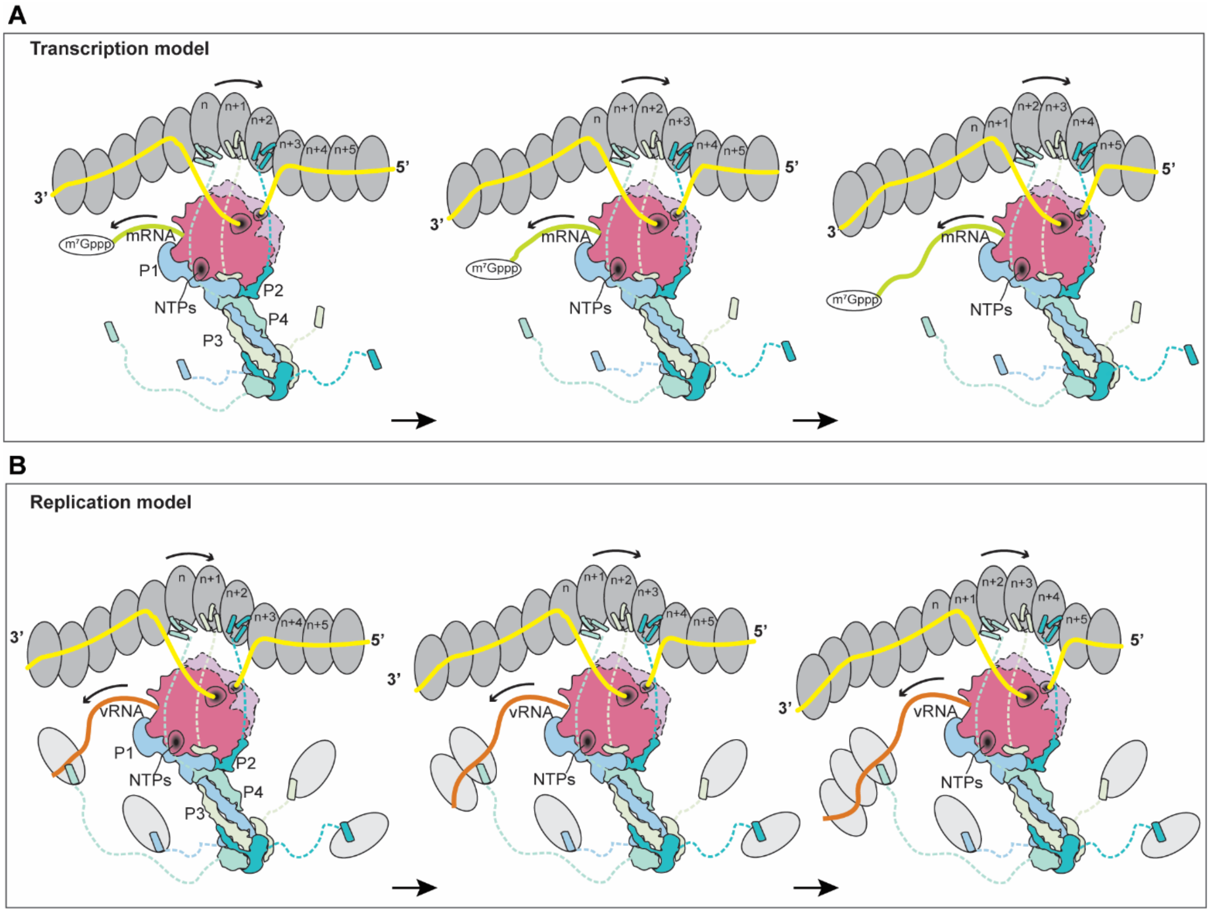

Appendix Figure S9. Cartoon for the sliding model for NiV RNA synthesis. The XD-domain of P1 interacts with the L-protein while the other XD-domains tether N-protein. Here, the L-P complex maintains continuous contact with the RNA template while advancing along its length.

A. During transcription, the synthesized mRNA is capped at the 5' end, and the cap is subsequently methylated.

B. During replication, the newly synthesized N-proteins are chaperoned by the N-terminal domain of the P-proteins and subsequently incorporated into the growing nascent RNA strand.

Appendix Table S1. Cryo-EM data collection, refinement and validation statistics

	NiV L-P complex (EMDB-50781), (PDB 9FUX)
**Data collection and processing**	
Magnification	165,000
Voltage (kV)	300
Electron exposure (e–/Å^2)	50
Defocus range (μm)	1.4 to 2.4
Pixel size (Å)	0.7303
Symmetry imposed	C1
Initial particle images (no.)	6,748,913
Final particle images (no.)	490,675
Map resolution (Å)	2.49
FSC threshold	0.143
Map resolution range (Å)	2.5 - 30.00
**Refinement**	
Initial model used (PDB code)	ModelAngelo
Model resolution (Å)	2.5
FSC threshold	0.5
Map sharpening *B* factor (Å^2)	85.1
Model composition	
Non-hydrogen atoms	14537
Protein residues	1811
Ligands	2
B factors (Å^2)	
Protein (min/max/mean)	16.19/191.32/61.92
Ligand (min/max/mean)	94.27/122.61/108.44
R.m.s. deviations	
Bond lengths (Å)	0.004
Bond angles (°)	0.596
Validation	
MolProbity score	1.63
Clashscore	7.32
Poor rotamers (%)	0.67
Ramachandran plot	
Favored (%)	96.53
Allowed (%)	3.41
Disallowed (%)	0.06

Appendix Table S2. X-ray data collection and refinement statistics.

	CD
**Data collection**	
Space group	P 1 21 1
Cell dimensions	
a, *b*, *c* (Å)	40.76, 50.54, 61.00
α, β, γ (°)	90.00, 95.71, 90.00
Resolution (Å)	60.80 - 1.85 (1.92 - 1.85)
R_{sym} or R_{merge}	0.102 (2.065)
$I / \sigma I$	10.3 (0.6)
Completeness (%)	91.30% (55.69%)
Redundancy	6.0 (3.3)
**Refinement**	
Resolution (Å)	60.80 - 1.85 (1.92 - 1.85)
No. reflections	19413 (1175)
R_{work} / R_{free} (%)	19.3 (33.1) / 21.9 (37.3)
No. atoms	2358
Protein	2146
Ligand/ion	3
Water	209
Average B-factor, all atoms (Å2)	28.8
R.m.s. deviations	
Bond lengths (Å)	0.0023
Bond angles (°)	0.55
Ramachandran Plot	
Favored (%)	98.44
Outliers (%)	0.00

Values in the parentheses are for the highest resolution shell.
