# Supplementary figures and images for: Structure of the Nipah virus polymerase complex
Source: EMBO J. 2024 Dec 30;44(2):563–86. doi: 10.1038/s44318-024-00321-z (PMC11730344; doi:10.1038/s44318-024-00321-z)

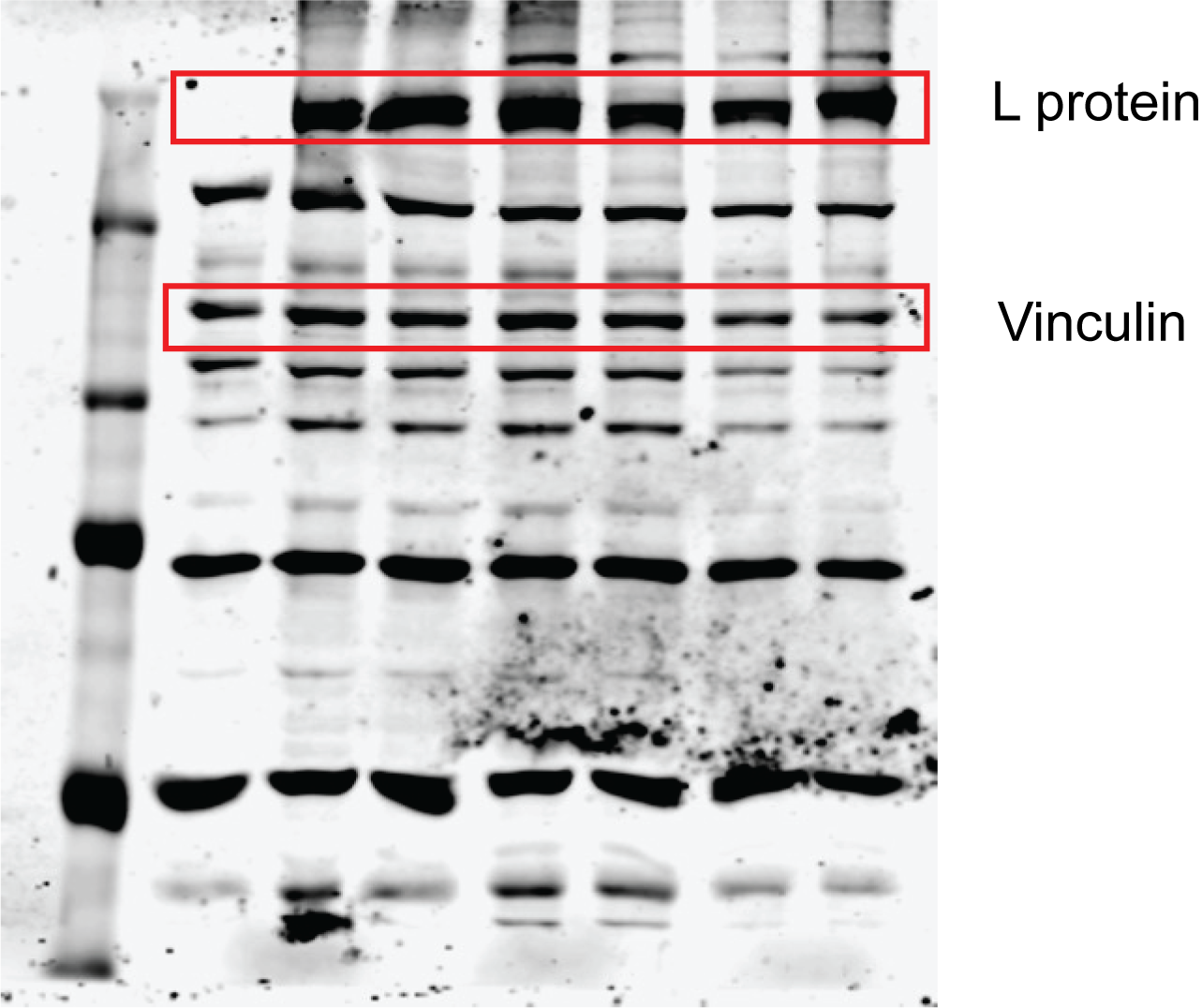

Supplement: Supplementary file 3 — Source data Fig. 6 [file 44318_2024_321_MOESM3_ESM.zip › SD_6E.tif]

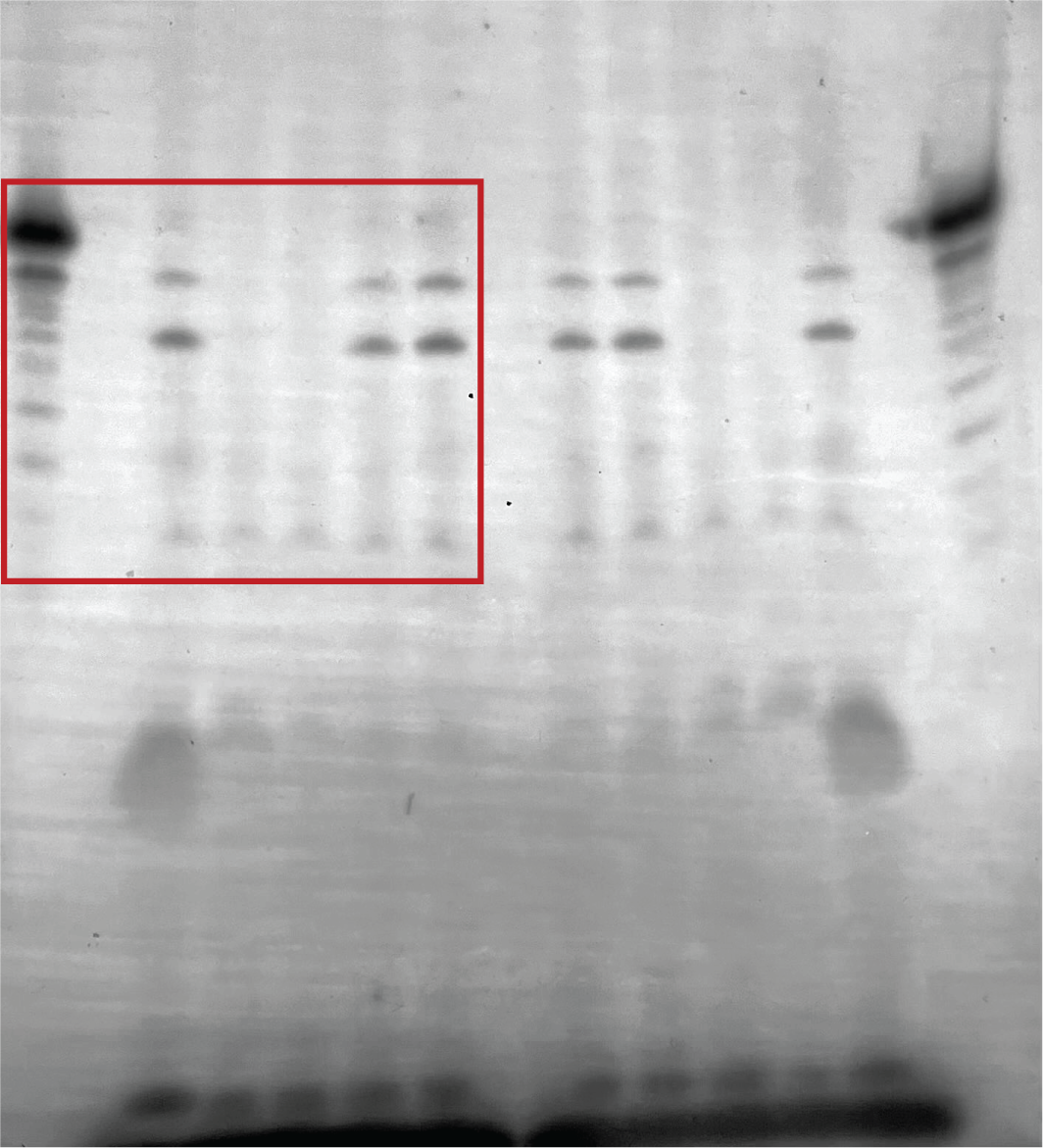

Supplement: Supplementary file 3 — Source data Fig. 6 [file 44318_2024_321_MOESM3_ESM.zip › SD_6F.tif]
