# Supplementary material for: Structure of the Nipah virus polymerase complex
Source: EMBO J. 2024 Dec 30;44(2):563–86. doi: 10.1038/s44318-024-00321-z (PMC11730344; doi:10.1038/s44318-024-00321-z)
Supplement: Supplementary file 5 — Expanded View Figures [file 44318_2024_321_MOESM5_ESM.pdf]

Expanded View Figures

Figure EV1. Purified NiV L-P complex is active.

(**A**) Size exclusion chromatography profile of the L-P complex. Fractions eluting during the second peak (indicated by an arrow) were collected for subsequent functional and structural studies. An SDS-PAGE analysis of the purified protein complex is shown to the left of the chromatogram. (**B**) SDS-PAGE analysis of purified L-protein mutants, including the active site mutant (D832A), HR mutant (HR-AA: H1347A, R1348A), GT mutant (GT-AA: G1273A, T1276A), and Mg1 mutant (Mg1-AAA: S1523A, N1526A, S1529A), are shown. (**C**, **D**) In vitro [α^{32}P]-GTP incorporation assays for the 3′ extension activity of the L-P complex assessing elongation with (+) or without (−) a 4-mer primer, a 12 nucleotide(nt)-long NiV leader sequence as a template, wild type NiV L-protein or the RdRp-domain active site mutant (L-D832A) (**C**). To evaluate the effect of remdesivir on RNA synthesis ATP was substituted with remdesivir triphosphate (**D**). A 12 nt sequence with a U-to-C mutation at position 5 (highlighted in orange) served as a template. A radiolabelled 12-mer RNA sequence served as a marker (M), lengths are indicated on the left. Source data are available online for this figure.

 © The Author(s)

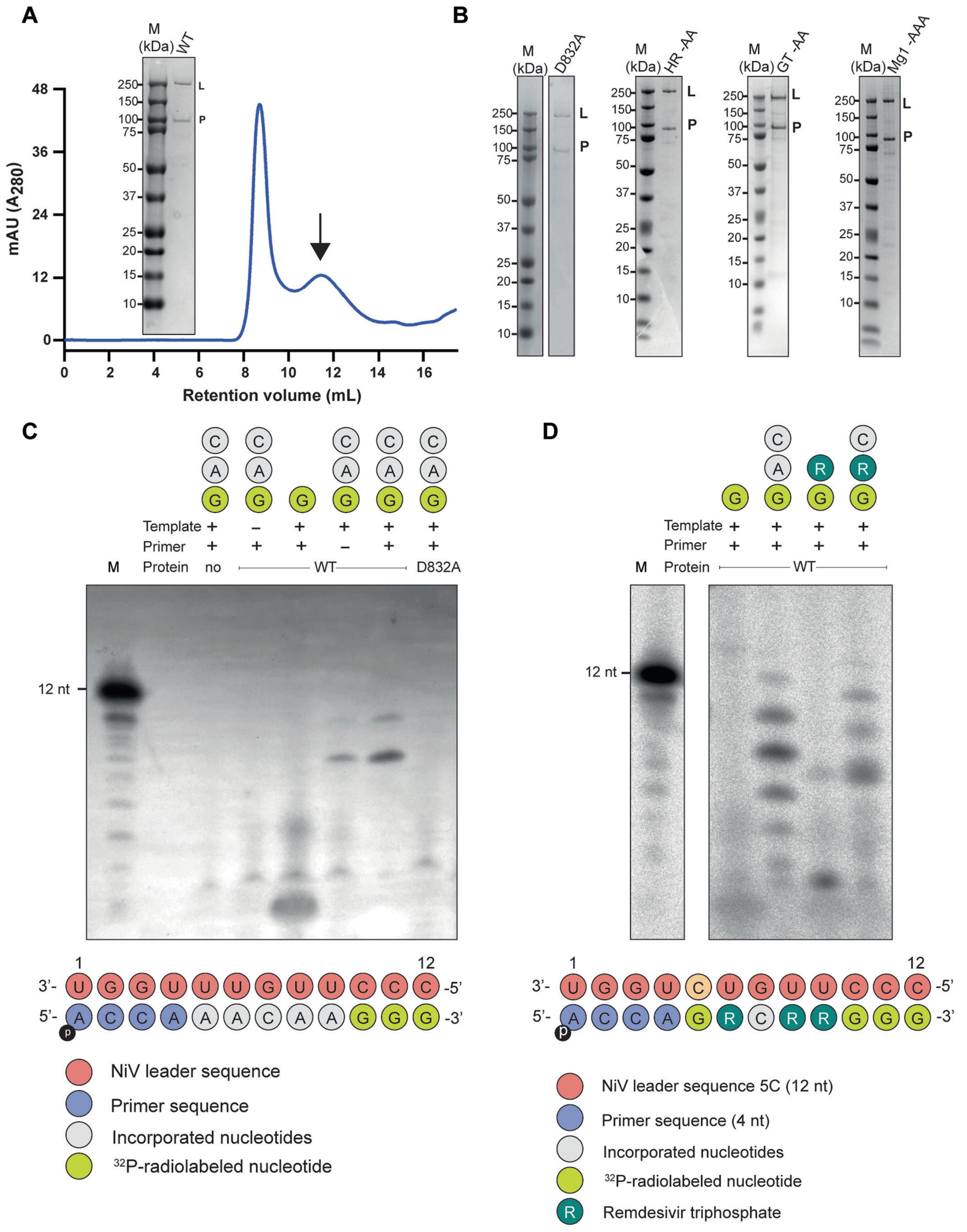
A
M (kDa)
WT
250
150
100
75
50
37
25
20
15
10
L
P
48
36
24
12
0
mAU (A280)
0 2 4 6 8 10 12 14 16
Retention volume (mL)
B
M (kDa)
D832A
HR -AA
GT -AA
Mg1 -AAA
L
P
C
Template + – + + + +
Primer + + + – + +
M
Protein no WT D832A
12 nt
1
12
3'- U G G U U U G U U C C C -5'
5'- A C C A A A C A A G G G -3'
p
NiV leader sequence
Primer sequence
Incorporated nucleotides
32P-radiolabeled nucleotide
D
Template + + + +
Primer + + + +
M
Protein WT
12 nt
3'- U G G U C U G U U C C C -5'
5'- A C C A G R C R R G G G -3'
NiV leader sequence 5C (12 nt)
Primer sequence (4 nt)
Incorporated nucleotides
32P-radiolabeled nucleotide
R
Remdesivir triphosphate

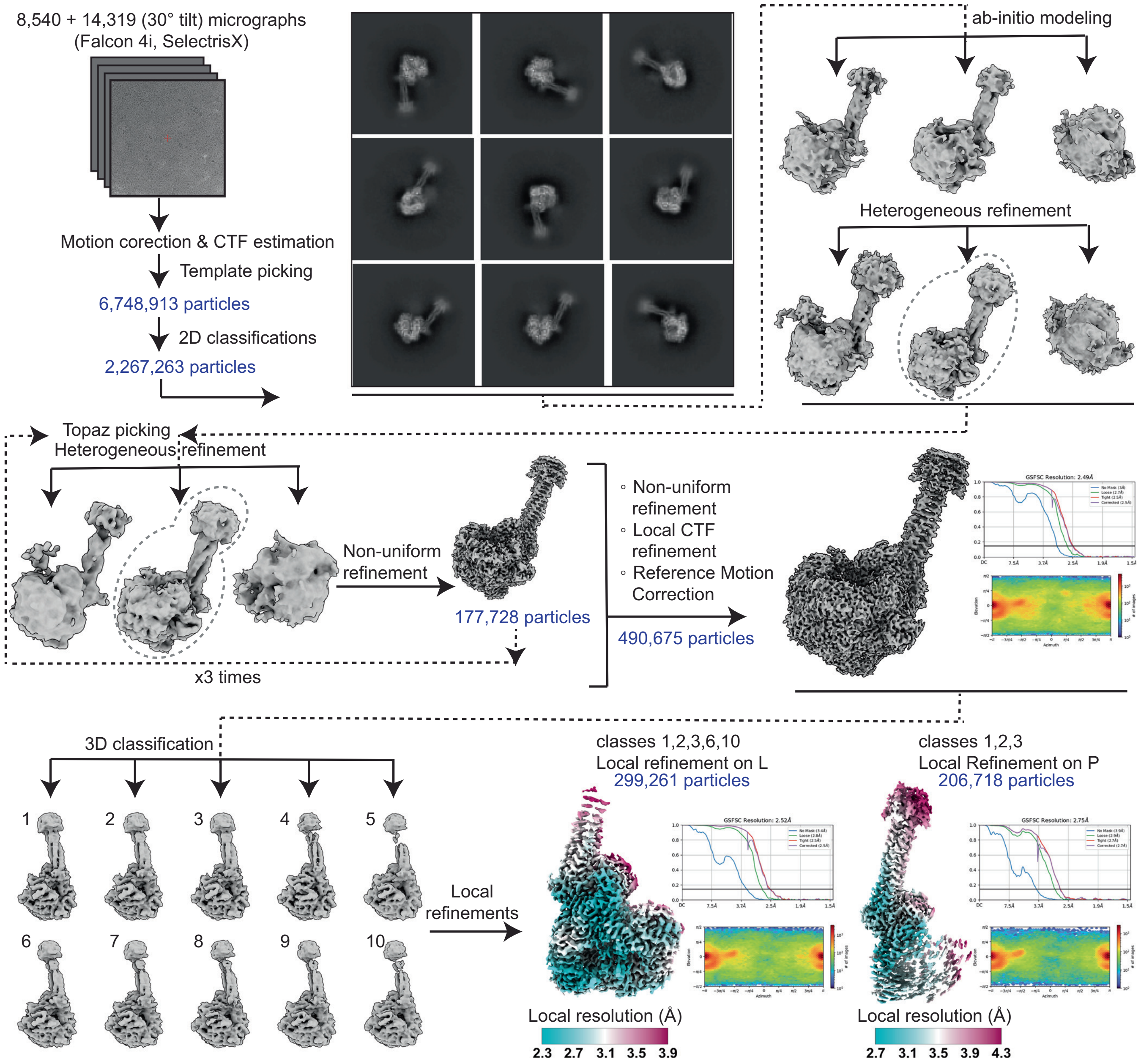

Figure EV2. Cryo-EM data processing workflow.

Flowchart indicates the path for data processing, classification and map reconstitution of the NiV L-P complex using CryoSPARC.

© The Author(s)

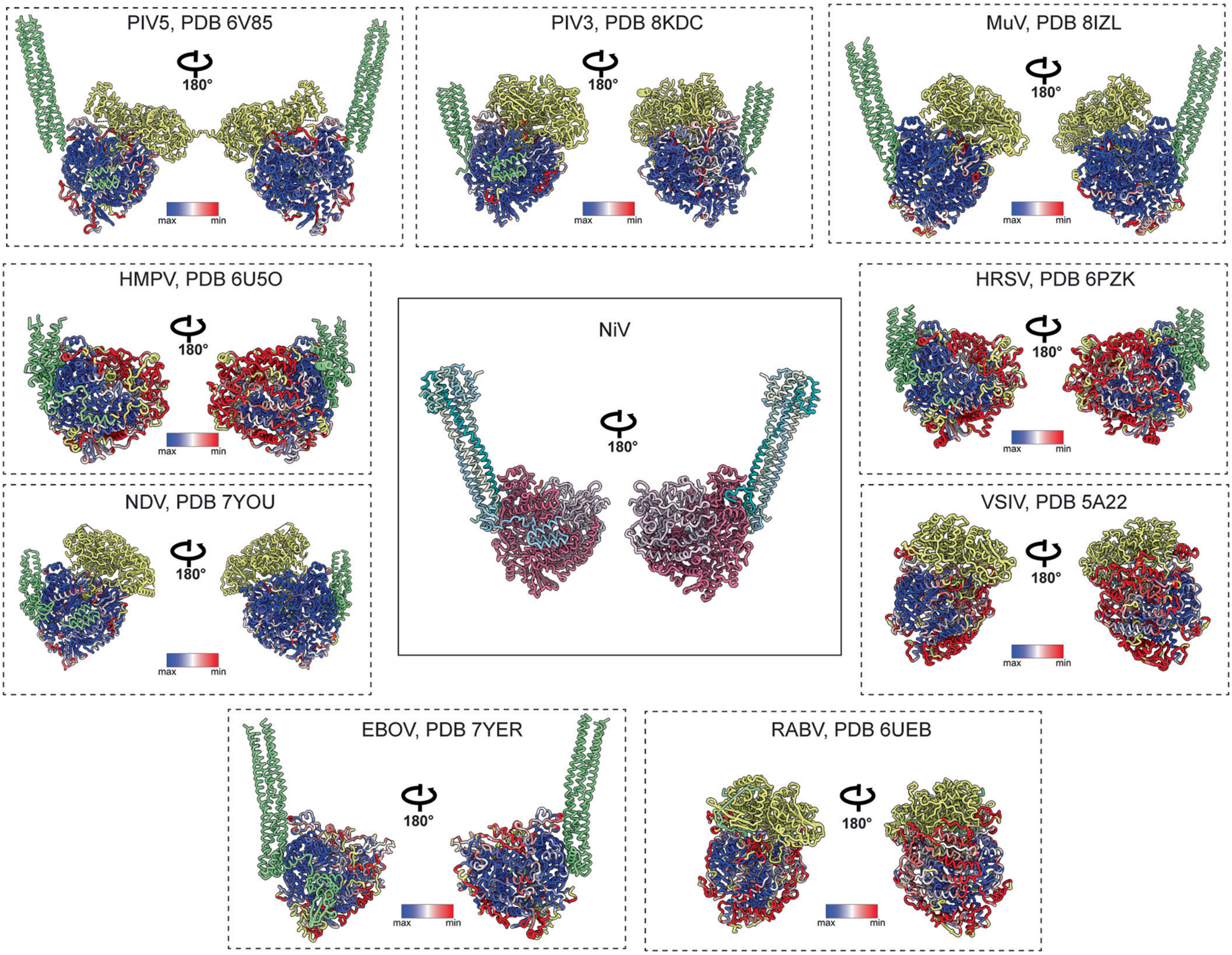

Figure EV3. Comparisons of the whole L-P complex with homologous structures.

The L-protein structure of NiV was aligned with homologous structures, including PIV5 (PDB 6V85, RMSD 1.223 Å), PIV3 (PDB 8KDC, RMSD 1.113 Å), MuV (PDB 8IZL, RMSD 1.182 Å), HMPV (PDB 6U50, RMSD 1.338 Å), NDV (PDB 7YOU, RMSD 1.134 Å.), EBOV (PDB 7YER, RMSD 1.349 Å), HRSV (PDB 6PZK, RMSD 1.372 Å), VSIV (PDB 5A22, RMSD 1.297 Å), and RABV (PDB 6UEB, RMSD 1.252 Å) The structures were superimposed onto the NiV L-protein, and the RMSD values were calculated in ChimeraX. The sequence alignments of the L-protein structures were colour-coded based on residue conservation, with blue representing highly conserved residues and red indicating the least conserved. The residues present in the homologous L-protein structures but absent in NiV L-protein structure were coloured in yellow. For simplicity, the P-proteins of the homologous structures were shown in pale green.

© The Author(s)

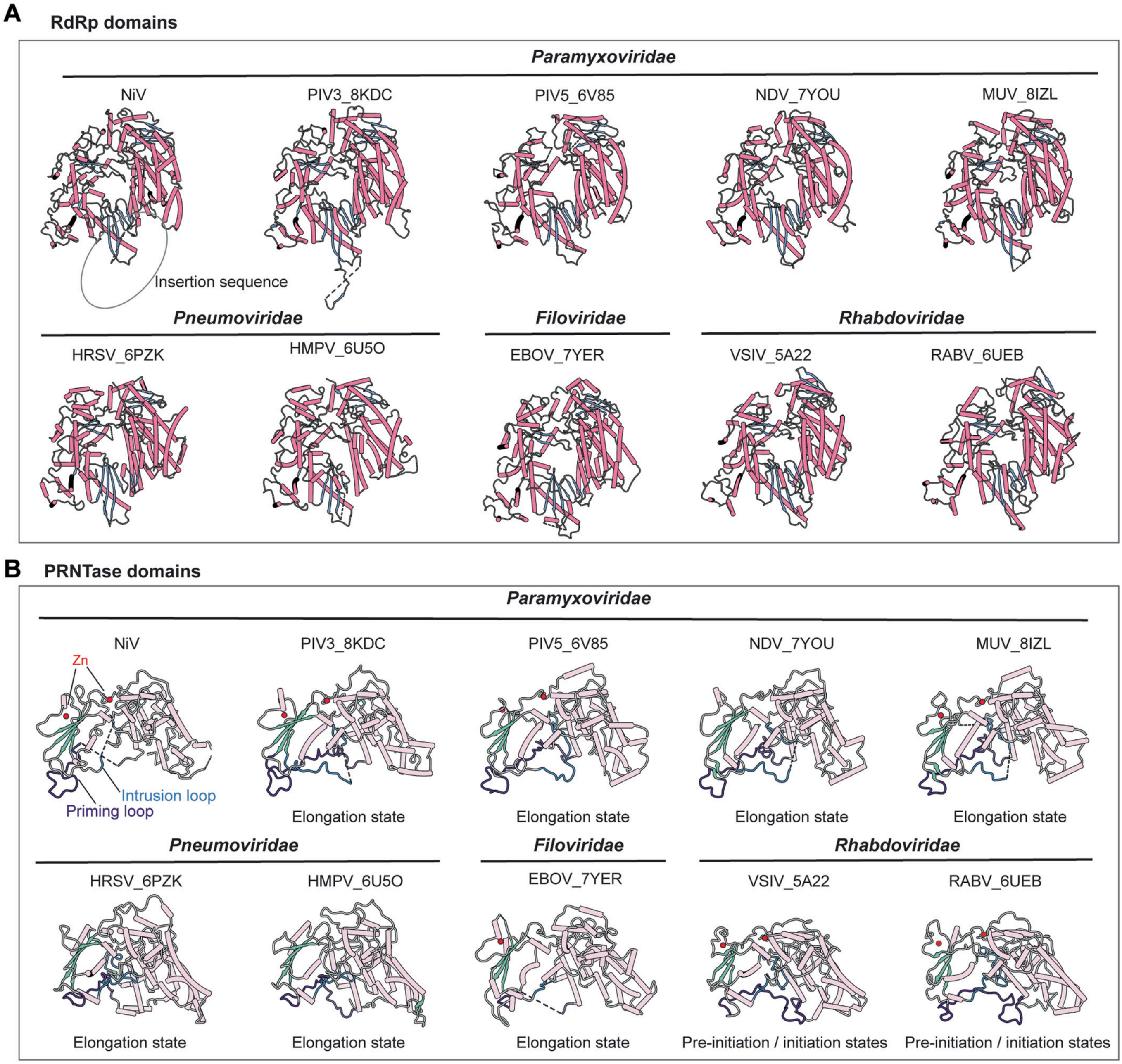

Figure EV4. Structural comparisons of RdRp- and PRNTase-domains of NiV L-protein with those of nsNSVs.

(**A**) The RdRp-domains are shown with helices coloured in pale purple red and β-sheets in blue. The location of the unmodelled palm-insertion sequence is shown by a dashed circle. (**B**) The PRNTase-domains are represented using secondary structure colourings: helices are shown in pale pink, β-strands are in green. Zinc ions bound to the PRNTase-domains are coloured in red, while the priming loop is represented in dark purple and the intrusion loop in marine blue.

 © The Author(s)
